# Case Report: Chronic Active Epstein–Barr Virus Infection With Subcutaneous Nodules and Systemic Damage

**DOI:** 10.3389/fmed.2022.759834

**Published:** 2022-03-31

**Authors:** Huating Luo, Zhe Yuan, Bo Qin

**Affiliations:** Department of Infectious Diseases, The First Affiliated Hospital, Chongqing Medical University, Chongqing, China

**Keywords:** chronic active Epstein-Barr virus infection, cutaneous chronic active EBV infection, EBV-associated hemophagocytic lymphohistiocytosis, allogeneic hematopoietic stem cell transplantation, EBER

## Abstract

Chronic active Epstein–Barr virus (CAEBV) infection is a rare and progressive systemic lymphoproliferative disorder often beginning as an infectious mononucleosis (IM)-like illness. It manifests with fever, splenomegaly, hepatitis, lymphadenopathy, and occasionally cytopenias, pneumonitis, and vasculitis. We report a 23-year-old woman with fever and subcutaneous nodules first appearing on the limbs and then spreading to the body. Peripheral blood EBV antibodies were elevated and EBV-DNA loads significantly increased. A skin and lymph node biopsy identified T cell-based lymphocyte infiltration and EBV-encoded RNA positivity (EBER+). CAEBV was finally diagnosed. During the illness, her disease progressed to hemophagocytic syndrome (HPS). The patient then successfully received an allogeneic hematopoietic stem cell transplantation (HSCT) at 6 months. Current follow-up at 2 years indicated a stable condition and six negative EBV-DNA tests, and we reviewed the clinical manifestations, mechanism, diagnosis and differential diagnosis, treatment, and prognosis of CAEBV. Finally, subcutaneous nodules may occur when CAEBV invades the skin; therefore, clinicians must identify the cause of these nodules early. HSCT is effective but its timing must be appropriate.

## Introduction

Chronic active Epstein–Barr virus (CAEBV) is a progressive disease characterized by systemic inflammation and the development of neoplasms which may lead to hemophagocytic syndrome (HPS) and lymphoma. CAEBV is considered a lymphoproliferative rather than infectious disease (1). This is a rare systemic EBV+ polyclonal, oligoclonal or monoclonal T cell, or NK cell lymphoproliferative disorder (LPD) that has a range in severity. Various clinical manifestations, fever, hepatosplenomegaly, systemic, lymphadenopathy, liver function damage, pancytopenia, and vasculitis are occurred (2). CAEBV severity is related to the host's immune response and EBV-DNA load (3). Notably, the infection typically involves multiple organ systems and presents with diverse clinical symptoms; fever is the most common whereas cutaneous lesions are rare. Typical cutaneous CAEBV manifestations are precipitated by mosquito bites (sMBA) or hydroa vacciniforme (HV) alterations (4). Currently, patients with CAEBV with subcutaneous nodules have not been reported. Here, we report a patient with fever with subcutaneous nodules who progressed to systemic CAEBV infection. A therapeutic approach was difficult due to the coexistence of viral infection and monoclonal T cell proliferation. During follow-up, HPS appeared and a timely allogeneic hematopoietic stem cell transplant (HSCT) was performed to facilitate remission. This rare case was reviewed in terms of clinical manifestations, differential diagnostics, pathological features, and current themes related to disease pathogenesis and management.

## Case Presentation

**Figure 1:**
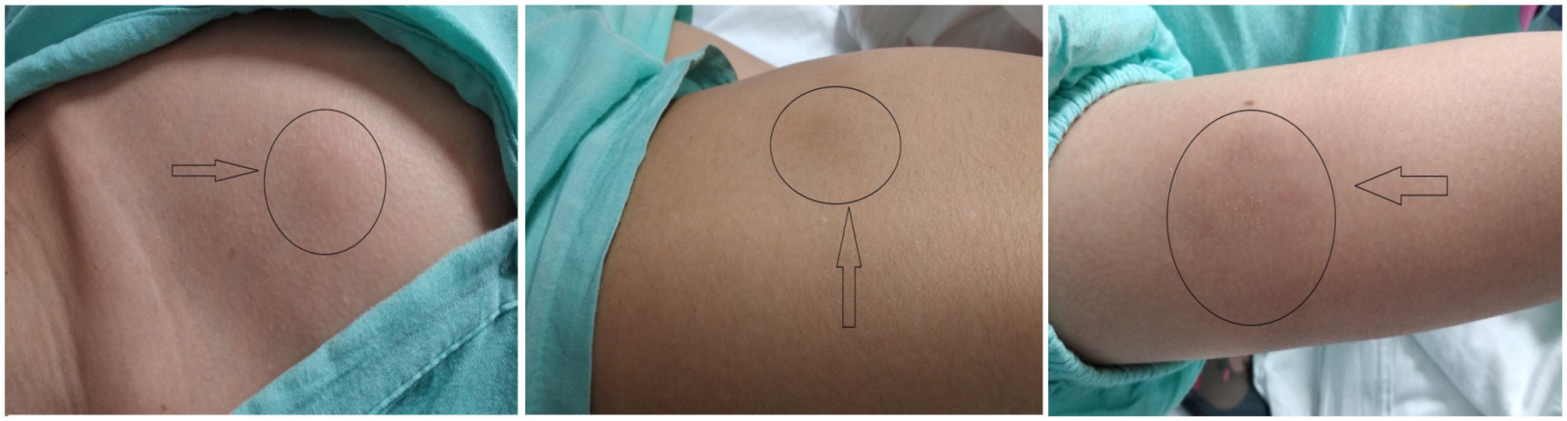
Characteristic clinical manifestations.

A 23-year-old-woman complained of recurring subcutaneous nodules for 8 years. She was aggravated and was accompanied by fever for more than 1 month before being admitted on May 15th, 2019 to our department. Eight years prior, the patient had observed for the first time several peanut-sized subcutaneous nodules on her left lower limb. The texture was hard and some nodules were tender. They disappeared ~1 month later without treatment. In the intervening 8 years, the subcutaneous nodules repeatedly reappeared after stressful episodes or colds. Nodule sizes varied, from peanut size to golf ball size, and the number of nodules gradually increased during the disease course. Nodules exhibited the following characteristics (Figure 1): (1) the limbs were mainly affected with variably sized nodules, (2) new nodules were tender and partially red in color, (3) nodules did not develop into blisters and ulcers and the patient never showed photosensitivity or hypersensitivity to mosquito bites, and (4) nodules completely disappeared after specific periods.

Two months before admission, nodule numbers increased, became enlarged, and appeared on the face, scalp, abdominal wall, and both lower limbs. The patient experienced fever for 1 month with no apparent cause. Her body temperature ranged between 38.5°C and 39.1°C. The fever type was irregular and was accompanied by fragile and occasional headaches, and she denied any history of chills, pharyngalgia, cough and expectoration, abdominal pain, diarrhea, and localized joint pain. Laboratory tests reported the following: serum alanine aminotransferase (ALT) 563 U/L; serum aspartate transaminase (AST) 433.7 U/L; total bilirubin (TBIL) 65.4 mmol/L, direct bilirubin (DBIL) 52.2 mmol/L. Blood test results were as follows: white blood counts (WBC) 3.95 × 10^9^/L; platelets 142.00 × 10^9^/L; and percentage of monocytes (MONO%) 13.4%; the spleen was enlarged with its lower edge 4 cm below the left costal margin. She tested negative for antigen and antibody markers for hepatotropic hepatitis A-E virus with the exception of hepatitis B surface antibody and had undetectable viral nucleic acid levels. B-ultrasound revealed splenomegaly. The patient's high fever still persisted after treatment with nonsteroidal antiinflammatory drugs at her local hospital. After this, she was referred to our hospital for further investigation. The patient had no history of drug allergies, photosensitivity, and mosquito bite allergies. Physical examination revealed multiple enlarged lymph nodes at the front and back of the neck, and a total of 17 subcutaneous nodules were located all over the body, the largest of which (5 cm × 4 cm) was located in the abdomen wall. The new nodules protruding from the skin surface exhibited clear boundaries, were hard in texture, and had poor mobility, local tenderness, and some redness.

**Figure 2:**
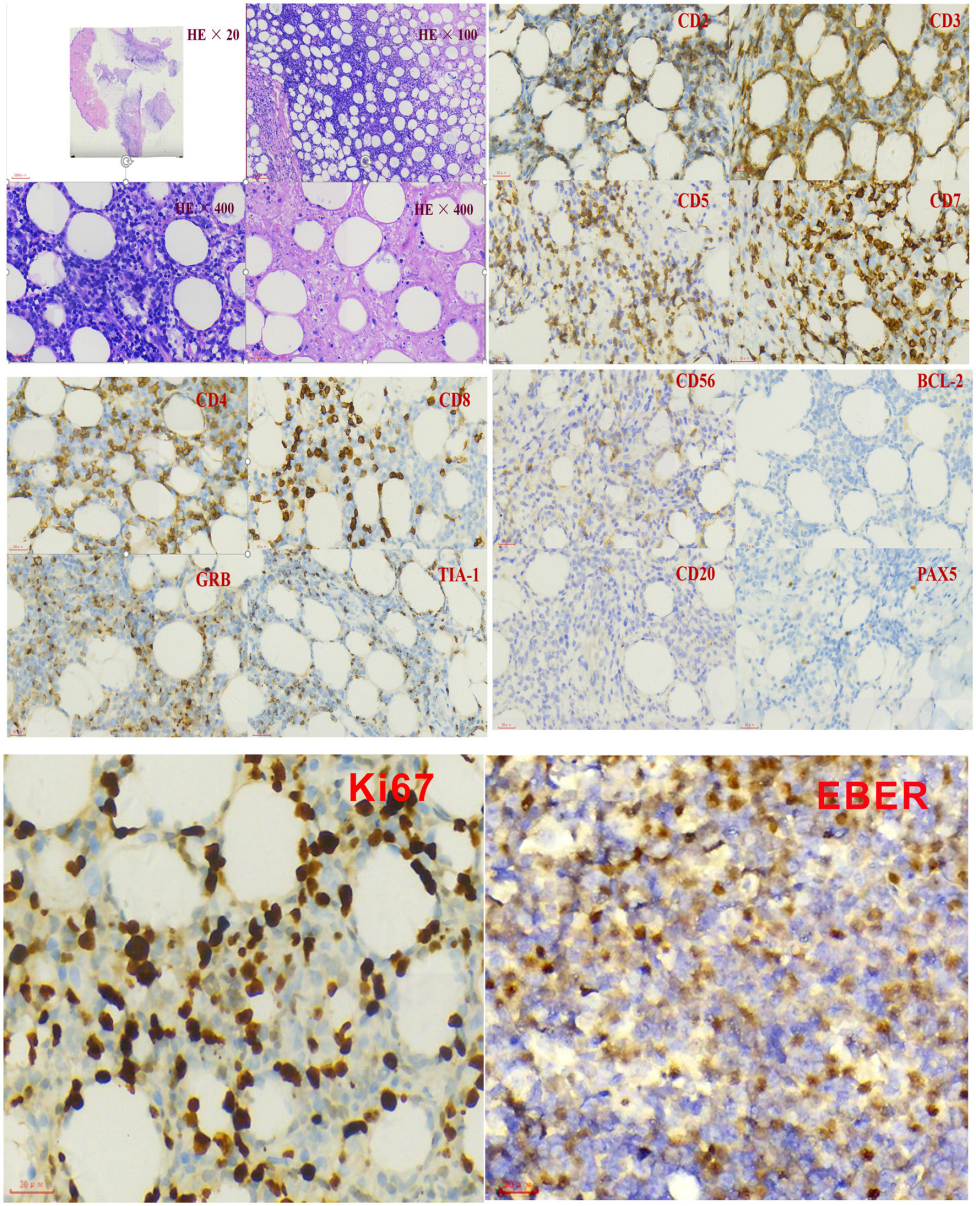
Immunohistochemistry and *in situ* hybridization with Epstein–Barr virus-encoded small NA (EBER) probe in the skin biopsy.

**Figure 3:**
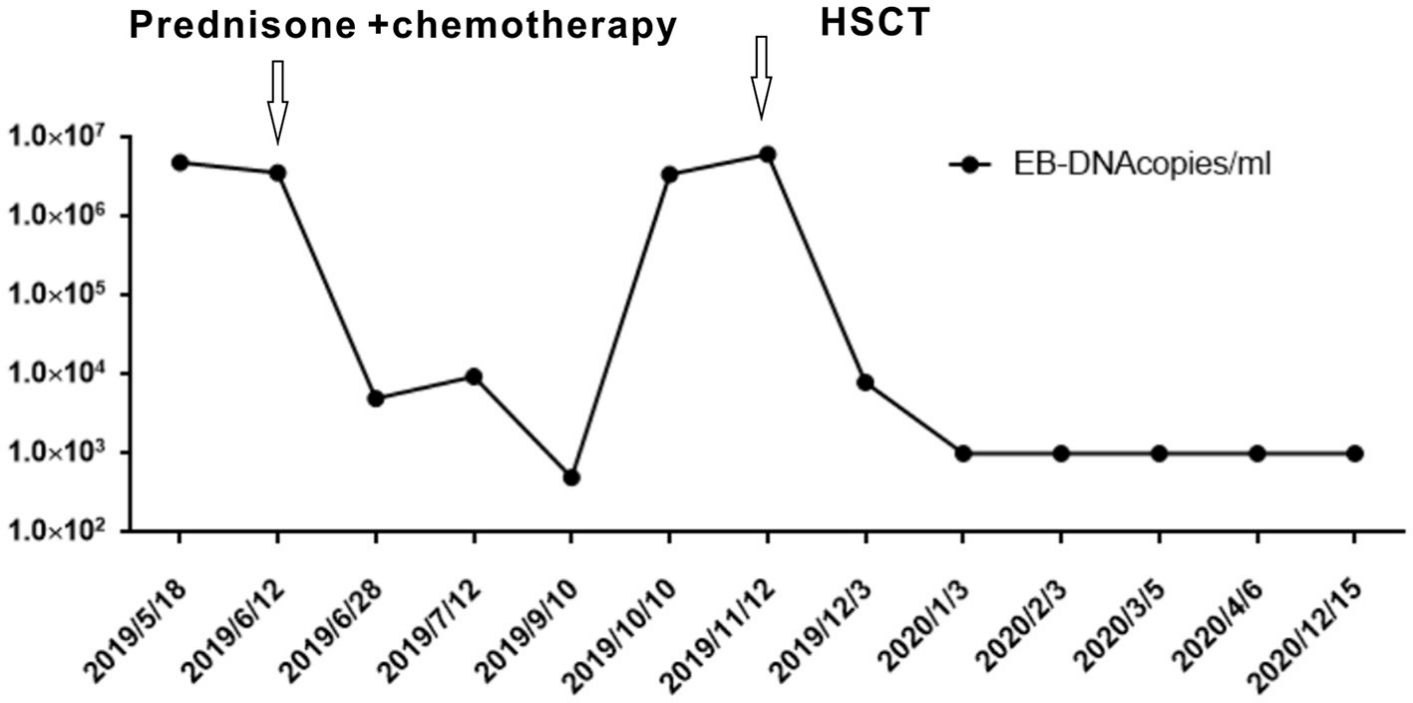
Epstein–Barr virus (EBV) viral load from 2019 to 2020.

Further blood routine examination reported that MONO% were elevated. Aspartate aminotransferase and alanine aminotransferase were moderately elevated. EBV antibody spectrum showed EBV-CA-IgG (+), EBV nuclear antigen (+), and EBV-early antigen (EA)-IgG (+). EBV-DNA levels were 1.42 × 10^6^ copies/ml; T cell subsets; total T lymphocyte count 391. Clusters of differentiation (CD) counts were as follows: CD4 (+) 21.05%, CD3 (+) CD4 (+) 8.83%, and CD3 (+) CD8 (+) 11.53%, and CD4 to CD8 ratio was 0.77%. B-ultrasound showed that superficial lymph nodes and the spleen were significantly enlarged. Thoracic, abdominopelvic computed tomography (CT) and bone marrow biopsy were normal. The patient was treated with nonsteroidal antiinflammatory drugs (NSAIDs), but the fever and subcutaneous nodules did not improve. Moreover, skin biopsy was performed and histopathological findings revealed large areas of necrosis in the skin and subcutaneous adipose tissue, with high levels of atypical T lymphocytes proliferating in the adipose septum around necrotic adipose tissue; EBER 30% (+) (>100/HPF). The immunohistochemical staining revealed that the infiltrate cells were positive for CD3, CD4, CD5, CD7, CD8, TIA-1, and GRB, but negative for CD2 and CD56. Approximately 50% cells expressed Ki-67 (Figure 2). Positive EBER signals were detected in skin biopsy specimens using *in situ* hybridization. The clinical manifestation, laboratory, and imaging examination were combined with pathological findings, and the final diagnosis was CAEBV with T cell proliferation as the dominant type (A1–A2 grade). One month after diagnosis, the patient was confirmed to progress with hemophagocytic syndrome. Treatment with oral prednisone was ineffective so the patient received chemotherapy 2 months after diagnosis of CAEBV. Six months later, she received a successful allogeneic HSCT. The patient was discharged and followed up for 2 years. Currently, the patient's condition is stable with six negative EBV-DNA tests (Figure 3). Her subcutaneous nodules disappeared, her body temperature remains normal, and no other complications occurred.

## Discussion

Ours is the first report of a patient with CAEBV with subcutaneous nodules. The patient's final diagnosis was T cell CAEBV. In the previous 8 years, she repeatedly experienced subcutaneous nodules and disease localized to the skin, in the absence of systemic symptoms. EBV infection in humans is usually asymptomatic and persists as a lifelong latent infection (5). Among patients with recurrent subcutaneous nodules over the past 8 years, she showed no signs of typical EB virus infection in the early stage of the disease, which is easy to be ignored by patients and clinicians. The disease develops rapidly in adults and progresses to multiple systems, accompanied by typical CAEBV clinical features such as fever, hepatitis, lymph node enlargement, and splenomegaly. The incidence of cutaneous lesion is very low and geographically distinct; the incidence in Asia is higher than Europe and the United States and is more common in children than adults (6). Pathologically, ulceration, bullae, and scar formation, with occasional necrosis symptoms, are indicated. Our patient was not a typical manifestation of CAEBV skin lesions. At first, we differentiated her condition from nodular panniculitis and subcutaneous panniculitis-like lymphoma. Although the age of 23 years at the time of consultation, patients often have a long history of recurrent skin lesions before systemic manifestations. The patient had already developed symptoms in childhood, which are characterized by subcutaneous nodules. These may be an initial clinical manifestation of CAEBV, with the progression to typical CAEBV system damage. Currently, reported CAEBV cases in children and adults are very rare (7); thus, this case highlights disease rarity and the importance of early recognition.

### Clinical Features

According to a 2016 World Health Organization (WHO) classification (8), CAEBV is defined as a disease of persistent inflammation with proliferating EBV-positive T or NK cells. During CAEBV, EBV-infected T or NK cells clonally proliferate and infiltrate into multiple organs leading to organ failure. These characteristics define CAEBV as a lymphoid neoplasm. However, the main symptom of CAEBV is inflammation; therefore, the condition exhibits two primary characteristics: systemic inflammation and neoplastic disease (1). This is because pathological injury involves almost all organ systems, which include lymph nodes, liver, spleen, blood, heart, and kidney, and cutaneous lesions (5). Persistent or intermittent fever, liver function abnormalities, hepatosplenomegaly, lymphadenopathy, and cytopenias are particularly common CAEBV manifestations. According to Kimura et al. (9), persistent fever occurs in 91% of patients with CAEBV, suggesting that CAEBV is a relevant condition when considering a differential diagnosis for fever of unknown origin. The incidence of CAEBV skin invasion is rare; especially in patients with subcutaneous nodules, which has not been reported, typical cutaneous lesion is mosquito bite allergy (33%), rashes (26%), and HV-like manifestations (10). Most patients have high antibody titers (EBV-VCA, EBV-EA) and high EBV viremia. Skin invasion in this patient can be regarded as a clinical manifestation of CAEBV, accompanied by fever, abnormal liver function, enlargement of spleen, continuous increase in blood EBV-DNA, and positive EBV antibody. Making a diagnosis depends on lymph node and tissue biopsy. In our case, skin and lymph node biopsies were EBER-positive, and EBV+ tumor cells were not seen.

A CAEBV prognosis is variable, with some patients who experienced a relatively indolent clinical course whereas others succumb to disease, an adverse prognostic feature is HLH or NK/T cell lymphoma (11). Our patient progressed slowly at first, then progressed rapidly when she experienced multiple system damage, and later complicated by HPS. Kimura et al. (1, 9) demonstrated that liver dysfunction, thrombocytopenia, fever (more than once a week), splenomegaly, anemia, and time from onset of more than 8 years are important poor prognostic predictors in patients with CAEBV. The prognosis for patients with T cell clonal proliferation is even worse than the NK cell type as reported by Cohen et al. in a retrospective analysis (12).

### CAEBV Diagnostics

When clinicians encounter patients with sustained fever, CAEBV as a differential diagnosis is rarely considered due to limited disease awareness and/or rarity. From our observations, the length of time from onset to diagnosis was 8 years. The recent persistent fever of the patient attracted attention and indicated disease progress. CAEBV diagnosis is based on clinical, laboratory, and biopsy findings (Table 1). If a patient with sustained inflammation of an unknown cause shows elevated anti-VCA-IgG or anti-EA-IgG antibodies and is positive for anti-VCA-IgA or anti-EA-IgA antibodies, CAEBV should be suspected. The next diagnostic step is the quantification of EBV-DNA loads in peripheral blood (PB) by polymerase chain reaction (PCR). Also, other EBV-DNA-positive diseases must be ruled out. EBV-DNA is usually detected in the serum of EBV-positive lymphomas, including, extranodal NK/T cell lymphoma, extranodal NK/T cell lymphoma (ENKL), EBV-positive Hodgkin lymphoma, and nasopharyngeal cell carcinoma (13). If EBV-infected cell infiltrating organ specimens are available, histological examinations using immune staining of our patient's lymph node and skin biopsies confirmed T cell infiltration and EBER positivity. When combined with her clinical manifestations and laboratory examinations, the patient was diagnosed with CAEBV; however, CAEBV rarely develops as solid tumors. In many cases, it is difficult to sample tissue biopsies in the clinic; therefore, PET/CT strategies are required for selected cases. Scans investigating hypermetabolic FDG foci in the liver, spleen, and bone marrow, and also multiple FDG-avid lymph nodes and skin, are highly suggestive of EBV-associated lymphoma (14). CAEBV lesions exhibiting FDG uptake are indicative of overt lymphoma development (15). When CAEBV is suspected, especially in case of a poor clinical and biological response to B cell depletion, symptom of CAEBV needs to be differentiated from other T or NK phenotype EBV+ lymphomas or LPD; before CAEBV was diagnosed, it is a challenge for pathologists, and comprehensive analysis should be conducted in combination with clinical and laboratory examination results.

**Table 1:** Diagnostic criteria of chronic active EBV infection of T and NK cell type (9).

Sustained or recurrent IM-like symptoms for >3 months, exclude primary EBV infection
Elevated EBV genome load in the peripheral blood (PB) (>102.5 copies/μg DNA)
Histologic evidence of infiltration of affected organs or PB by EBV+ lymphocytes
Exclusion of other possible diagnoses includes the following:
Congenital immune deficiencies, HIV infection, Iatrogenic immunosuppressive therapies, autoimmune or collagen vascular diseases, pathologic evidence of another malignant lymphoma (classic Hodgkin lymphoma, extranodal NK/T cell lymphoma, nasal type, peripheral T cell lymphomas, aggressive NK cell leukemia)

### CAEBV Treatment and Prognosis

Chronic active Epstein–Barr virus carries a risk of systemic disease development, progression to aggressive T and/or NK cell lymphomas, or HPS. Currently, several therapies are used to treat CAEBV, which include antiviral, chemotherapeutic, and immunomodulatory drugs, but with limited success. According to the study by Kimura et al. of 108 cases (16), of which the age of patients ranges from 1 to 50 years old with the median observation period of 46 months, 44% of the cases resulted in death due to severe organ dysfunction. The only effective treatment strategy is to eradicate EBV-infected T or NK cells using allogeneic HSCT. This approach is a radical treatment modality for CAEBV, but not only eliminates EBV-positive cells but also helps reestablish anti-EBV-specific cellular immunity in the recipient (17) as seen in our patient (Figure 3). Although a consensus exists that early HSCT produces better results, the decision to have an HSCT is often difficult, especially when the patient is stable without severe symptoms. With an increased understanding of CAEBV, an early diagnosis and combined treatments can improve patient outcomes.

## Conclusions

Chronic active Epstein–Barr virus is a rare disease represented by heterogeneous symptoms and inflammation over months to years. Clinicians need to be aware of the rare clinical manifestations of CAEBV. In this case, patient with CAEBV suffered from subcutaneous nodules and systemic damage for many years, and tissue biopsies are performed necessarily (such as bone marrow, spleen, skin, or liver). When a clinician doubts CAEBV, EBV must be tested for using *in situ* hybridization and EBV-DNA testing to facilitate prompt and appropriate therapy.

## Data Availability Statement

The original contributions presented in the study are included in the article/supplementary material, further inquiries can be directed to the corresponding authors.

## Ethics Statement

Written informed consent was obtained from the individual(s) for the publication of any potentially identifiable images or data included in this article.

## Author Contributions

All authors listed have made a substantial, direct, and intellectual contribution to the work and approved it for publication.

## Funding

This work was supported by General Project of Chongqing Natural Science Foundation (Grant No. csct2020jcyj-msxmX0221).

## Conflict of Interest

The authors declare that the research was conducted in the absence of any commercial or financial relationships that could be construed as a potential conflict of interest.

## Publisher's Note

All claims expressed in this article are solely those of the authors and do not necessarily represent those of their affiliated organizations, or those of the publisher, the editors and the reviewers. Any product that may be evaluated in this article, or claim that may be made by its manufacturer, is not guaranteed or endorsed by the publisher.
